# Depth-dependent effects of culling—do mesophotic lionfish populations undermine current management?

**DOI:** 10.1098/rsos.170027

**Published:** 2017-05-24

**Authors:** Dominic A. Andradi-Brown, Rachel Grey, Alicia Hendrix, Drew Hitchner, Christina L. Hunt, Erika Gress, Konrad Madej, Rachel L. Parry, Catriona Régnier-McKellar, Owen P. Jones, María Arteaga, Andrea P. Izaguirre, Alex D. Rogers, Dan A. Exton

**Affiliations:** 1 Department of Zoology, University of Oxford, The Tinbergen Building, South Parks Road, Oxford, OX1 3PS, UK; 2 Operation Wallacea, Wallace House, Old Bolingbroke, Spilsby, Lincolnshire PE23 4EX, UK; 3 College of Charleston, 205 Fort Johnson Road, Charleston, SC 29412, USA; 4 Whaleshark and Oceanic Research Center, Main Street, Utila, Bay Islands 34201, Honduras; 5 Bay Islands Conservation Association—Utila Chapter, Airport Road, Utila, Bay Islands 34201, Honduras

**Keywords:** mesophotic coral ecosystem, lionfish, pterois, ontogenetic migration, mesoamerican barrier reef, Utila Honduras

## Abstract

Invasive lionfish (*Pterois volitans* and *P. miles*) have spread widely across the western Atlantic and are recognized as a major threat to native marine biodiversity. Although lionfish inhabit both shallow reefs and mesophotic coral ecosystems (MCEs; reefs from 30 to 150 m depth), the primary management response implemented by many countries has been diver-led culling limited to reefs less than 30 m. However, many reef fish undergo ontogenetic migrations, with the largest and therefore most fecund individuals found at greatest depths. Here, we study lionfish density, body size, maturity and dietary patterns across the depth gradient from the surface down to 85 m on heavily culled reefs around Utila, Honduras. We found lionfish at increased densities, body size and weight on MCEs compared with shallow reefs, with MCEs also containing the greatest proportion of actively spawning females, while shallow reefs contained the greatest proportion of immature lionfish. We then compared lionfish behaviour in response to divers on shallow culled and mesophotic unculled Utilan reefs, and on shallow unculled reefs in Tela Bay, on the Honduran mainland. We found that mesophotic lionfish exhibited high alert distances, consistent with individuals previously exposed to culling despite being below the depth limits of removal. In addition, when examining stomach content, we found that fish were the major component of lionfish diets across the depth gradient. Importantly, our results suggest that despite adjacent shallow culling, MCEs retain substantial lionfish populations that may be disproportionately contributing towards continued lionfish recruitment onto the shallow reefs of Utila, potentially undermining current culling-based management.

## 1. Introduction

Lionfish, native to the Indian and Pacific Oceans and Red Sea, were first recorded in the western Atlantic in the 1980s, and have since become a major invasive species [1,2]. Two lionfish species have been recorded in this region, *Pterois volitans* and *P. miles*, though it is believed that *P. volitans* is responsible for much of the invasion, with *P. miles* mostly restricted to the waters around the US mainland [3]. On western Atlantic shallow reefs, lionfish have been reported 1.5 times larger and three times heavier than in their native range [4]. Lionfish are carnivores, and their arrival on shallow patch reef systems has been associated with declines in native fish recruitment of up to 79% [5] and prey fish declines of up to 65% [6]. The response to the invasion, in many locations, has been the introduction of lionfish culling programmes, where volunteer divers use handheld spears to remove lionfish from the reef [7]. Culling programmes in some areas have been effective in reducing lionfish densities [8], but as they are a highly fecund species, culling rates must remain high or populations will quickly recover [9].

Mesophotic coral ecosystems (MCEs; reefs from 30 to 150 m depth) are highly understudied [10], with most of the limited research focused on their native biodiversity [11]; however, a recent review has highlighted the potential threat posed to MCEs by invasive species [12]. Lionfish (*P. volitans*) now appear to have widely invaded MCEs throughout the western Atlantic region [2,13–15]. This MCE invasion by lionfish is unsurprising, as they have been reported on MCEs in several locations across their native range including in the Red Sea [16], the Philippines and Micronesia [15], New Caledonia [17] and American Samoa [18]. While lionfish densities in the invaded range are much higher than in the native range [4], their relative abundance across adjacent shallow reef to MCE gradients appear similar between invaded and native range sites [15]. In contrast with shallow reefs, the impacts of invasive lionfish on MCEs are poorly understood, though there is evidence that their consumption of fish trophic groups such as herbivores can lower grazing pressure on algal communities, leading to algal-dominated reefs through algal suppression of hard corals [13,19].

The extent to which lionfish in the invaded western Atlantic exhibit specific recruitment and ontogenetic migration between habitats is unclear. Analysis using proxy measures for maturity, such as fish weight or length, have suggested that lionfish ontogenetic migrations occur between shallow marine habitats and reef slopes (less than 30 m) [20]. Previous studies investigating whether lionfish ontogenetic migrations extend onto MCEs have relied on similar proxy measures for maturity, and found mixed results; for example in the Leeward Antilles, some locations had heavier lionfish on deeper reefs than shallow reefs, while other locations showed no correlation between depth and weight [21]. A recent meta-analysis across the western Atlantic identified lionfish with larger body lengths on MCEs than shallow reefs in three countries; The Bahamas, Curaçao and Honduras, with one country showing no relationship and another, which lionfish had recently colonized, displaying the opposite trend [15]. Therefore, it is not clear whether the previously documented ontogenetic migration to shallow-reef slopes extends to MCEs, and no studies have directly assessed lionfish maturity across shallow to MCE gradients.

As most lionfish culling in the western Atlantic is conducted by volunteer recreational divers, removals are generally limited to less than 30 m, with the majority concentrated even shallower. MCEs therefore have the potential to act as lionfish refuges in the presence of culling on shallow reefs, undermining management efforts [9]. This refuge effect could act through several non-mutually exclusive mechanisms, including: (i) large lionfish densities remaining on MCEs following shallow-reef-focused lionfish culling, and/or (ii) larger, and therefore more fecund, lionfish present on MCEs than shallow reefs which could be disproportionately responsible as a source of new lionfish recruits.

The presence of spearfishing is known to affect fish behaviour, leading to greater avoidance of fishers in areas where regular spearfishing occurs [22,23]. On shallow patch reefs, regular culling causes lionfish to react to approaching divers at greater distances [23]. This suggests that if MCE lionfish populations represent ontogenetic migration extensions, individuals will previously have been exposed to culling while in the shallows. Therefore, despite being below the culling depth limits, culling-induced behavioural changes may persist within MCE lionfish populations. By contrast, if MCE lionfish represent separate populations recruited onto the reef, they would be expected to exhibit behavioural responses similar to populations without culling.

In this study, we surveyed lionfish populations on shallow reefs and MCEs down to 85 m depth around Utila Island and on shallow reefs (less than 20 m) in Tela Bay, both in Honduras on the southern Mesoamerican Barrier Reef, Caribbean. Around Utila, we measured lionfish density on the reef, and speared lionfish to conduct detailed fish measurements and dissection, including lionfish maturity directly from the gonad developmental stage. We specifically tested if: (i) lionfish densities remain high on MCEs despite adjacent shallow-reef culling and, (ii) lionfish are more mature at increased depths, either of which would suggest a refuge role for MCEs enabling invasive lionfish to persist despite local management interventions. We recorded lionfish alert distance on Utilan culled shallow reefs and unculled MCEs, and Telan unculled shallow reefs. This allowed us to further support our results by testing whether: (iii) lionfish on MCEs exhibit behavioural responses to divers more similar to shallow lionfish populations without previous culling exposure than to shallow populations which have experienced intense regular culling.

## 2. Material and methods

### 2.1. Study site

**Figure 1.**
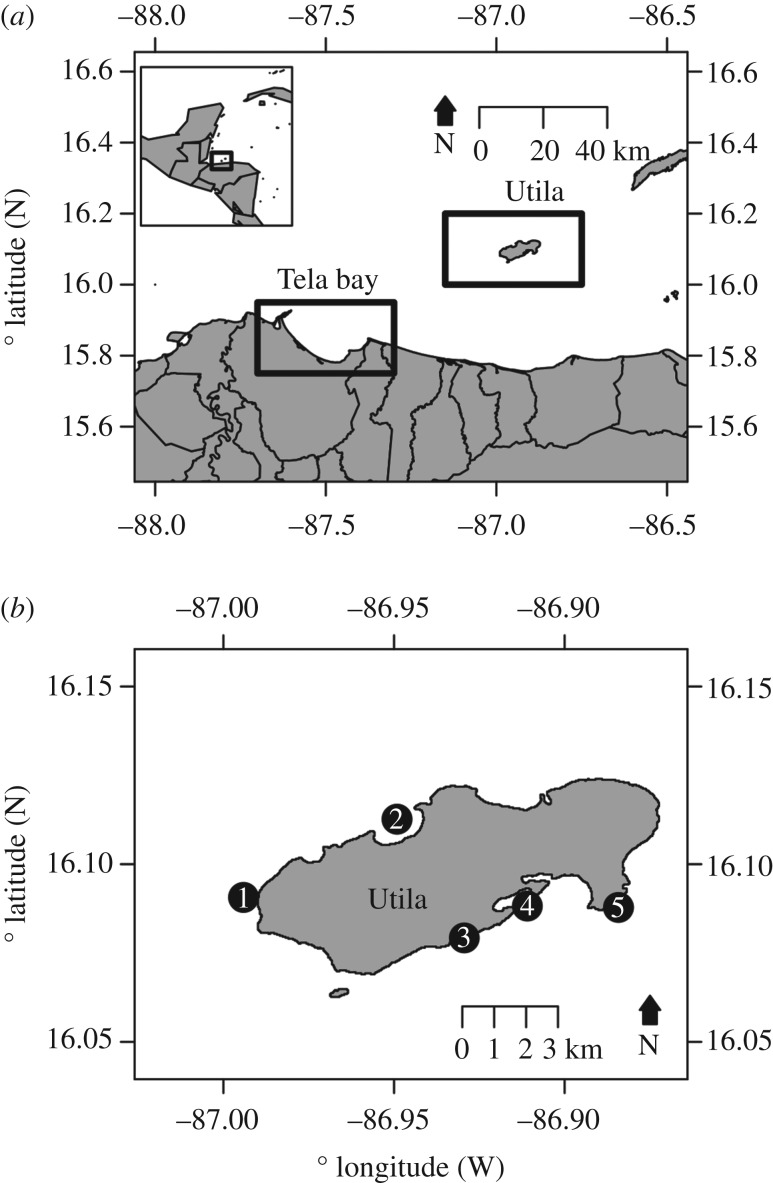
Map of study sites around Honduras. (*a*) The location of Utila Island and Tela Bay are indicated within boxes relative to the north shore of Honduras. Inset map shows the location of Utila and Tela relative to the western Atlantic region. Lionfish were speared to study their condition and diet around Utila only, while behavioural data were collected from both Utila and Tela. Around Utila culling is regularly conducted on all shallow fringing reefs, with adjacent MCEs unculled, while Tela shallow reefs are unculled. (*b*) Lionfish abundance surveys were only conducted in Utila at the marked sites, with numbers indicating sites as follows: (1) Raggedy Cay, (2) The Maze, (3) Little Bight, (4) Coral View, (5) Rocky Point.

Lionfish density and behaviour surveys alongside collections were conducted around Utila Island, Honduras on the southern Mesoamerican Barrier Reef (figure 1*a*). Utila is located within the Bay Islands National Marine Park, and is surrounded by fringing coral reefs. On the south shore, shallow reefs exist as a spur and groove system, sloping to approximately 30–40 m where the seabed flattens and an MCE patch reef system exists. On the north shore reefs are characterized by steep walls dropping to more than 100 m, with several narrow ledges approximately 20 m wide at various depths, depending on the site. Off the southwest of Utila, there are a series of cays and extensive offshore reef banks rising from the seabed with established shallow reef and MCE communities. Utila has a large number of dive centres (more than 10), resulting in year-round high lionfish culling intensity maintained on Utilan shallow reefs (shallow culled), yet little removal occurs from Utilan MCEs (mesophotic unculled).

To understand how continued culling alters lionfish behaviour, we conducted further lionfish behavioural surveys on the shallow (less than 20 m) reefs of Tela Bay (shallow unculled). Tela Bay consists of fringing reefs and the Banco Capiro reef system, an extensive reef bank located approximately 8 km offshore in the bay [24]. In contrast with Utila, the reefs of Tela have low levels of dive tourism, with only one seasonally operating dive centre. Therefore, lionfish are rarely culled on the reefs of Tela. Lionfish were first reported on the Caribbean coast of Honduras in May 2009 with numerous sightings throughout late 2009 and 2010 [2], followed by widespread colonization of reef habitats.

### 2.2. Lionfish density

Lionfish density was surveyed during July–September 2015 using video transects with a diver-operated stereo-video system [25]. Four transects were conducted at each depth, using the following depths: 5, 15, 25, 40, 55, 70 and 85 m at five sites around Utila (figure 1*b*, see electronic supplementary material, 1 for GPS coordinates). Transects were 50 m long and 5 m wide, and completed to a maximum depth of 40 m at Little Bight and Coral View, 55 m at Raggedy Cay and Rocky Point and 85 m at The Maze. These maximum survey depths represent the maximum depth of each site, with the exception of Raggedy Cay and The Maze, where they represent the approximate depth limit of light-dependent scleractinian corals based on preliminary dive surveys. All transects were carried out by divers using Hollis Prism 2 (Hollis, San Leandro, CA, USA) or rEvo X micro (rEvo rebreathers, Bruges, Belgium) closed-circuit rebreathers [26]. Transects were analysed using EventMeasure software (v. 3.51, SeaGIS, Melbourne, Australia), with boundaries defined 2.5 m on either side of the transect tape, giving the total number of lionfish per 250 m^2^ survey area for each transect.

### 2.3. Lionfish condition and diet

To enable the detailed study of changes in lionfish biology across the depth gradient, we used data collected from lionfish hand-speared by divers around Utila from the period 2014–2016 (see electronic supplementary material, 2 for detailed lionfish numbers from different sources). The depth of collection was recorded for all Utilan lionfish within three depth intervals (numbers of speared lionfish given in brackets for each depth interval); shallow: 0–25 m (*n* = 1049), intermediate: 25–40 m (*n* = 188) and MCE: more than 40 m (*n* = 155). The high number of lionfish speared from the shallow reefs reflects the large number of divers searching shallow reefs to cull lionfish, while the deeper reef lionfish collections have fewer speared lionfish because they came from research activities specifically targeting below the normal culling limit. Therefore, differences in the number of speared lionfish between depths should not be considered an indication of differing lionfish densities at the different depth zones.

All collected lionfish were dissected following the standardized dissection techniques outlined by Green *et al*. [27]. In summary: recording total length (length from the tip of the snout to the end of the caudal fin), weight, sex and gonad weight. Female gonads were staged on a five-level score as follows: (i) immature, (ii) early developing, (iii) developing, (iv) spawning capable and (v) actively spawning. Male lionfish gonads were not staged because of the difficulties in distinguishing between immature and spawning capable. See Green *et al*. [27] for images and detailed descriptions of each gonad stage. Fat tissue was visually identified and removed by hand from around internal organs and weighed. The gonad:body weight ratio was calculated as the gonad weight divided by the lionfish body weight, and the proportion of lionfish body fat was calculated as the fat tissue weight divided by the lionfish body weight for each lionfish. Lionfish stomachs were opened and the contents were counted and identified in broad categories: fish, shrimp, crab, other invertebrates and algae. If possible, consumed fish were identified to family level.

### 2.4. Lionfish behaviour

During 2015 and 2016, we opportunistically conducted additional shallow reef (Utila and Tela) and MCE (Utila) roving lionfish searches to gather data on lionfish alert distances based on Côté *et al*. [23]. Alert distance represents the distance at which a diver can approach a lionfish before it responds to the diver's presence [23]. Alert responses can be variable including retreating from the diver into reef structure, or turning the body and fanning fins. During these roving search dives, dive teams swam along the reef within the shallow reef or MCE zone looking for lionfish on the reef surface and within reef crevices and areas of complexity. All surveys were conducted during daylight hours between 07.30 and 16.30. To measure the alert distance, a diver slowly approached the lionfish with their spear ready and extended towards the lionfish. The diver watched the lionfish's behaviour, and estimated the distance in centimetres from the spear tip to the lionfish when the lionfish first reacted to the diver's approach. The lionfish was then speared, the depth recorded and the individual dissected to incorporate into the wider dataset. To maximize accuracy, surveyors practised distance estimation underwater between objects separated by known distances.

### 2.5. Data analysis

To identify changes in lionfish length and weight distribution with depth, we used a pairwise Kolmogorov–Smirnov test with a correction for multiple comparisons following the false discovery rate [28] based on three depth categories: 0–25, 25–40 and more than 40 m. To understand changes in lionfish populations with depth, we plotted the proportion of female lionfish at each maturity stage by depth band, and used Analysis of Variance (ANOVA) to compare the gonad:body weight ratio and fat:body weight ratio across the depth gradient allowing us to control for differences in lionfish weight on gonad and body fat changes. We assessed differences between groups using Tukey's honest significant differences (THSD), considering results significant if *p *< 0.05. To further ensure that patterns in the proportion of body fat and maturity were not driven by changes in body size, we tested for relationships between lionfish length and proportion of body fat using analysis of covariance (ANCOVA). For female lionfish, we tested for a relationship between gonad weight and the proportion of body fat while controlling for maturity level, fish weight and depth. Prior to analysis, datasets were tested and did not deviate significantly from the assumptions required by the ANOVA/ANCOVA.

To identify differences in the feeding rates at different depths, we divided lionfish stomachs into successes (food items present) and failures (food items absent), and fitted a binomial generalized linear model (GLM) with a logit link function. We examined patterns in lionfish prey consumption across the depth bands by plotting the proportion of different broad diet categories (fish, shrimp, crab, other invertebrates) based on aggregating all stomach items recorded in lionfish within a depth band. We further examined changes in prey fish consumption with depth based on dividing lionfish into four body size groupings (less than 150, 150–250, 250–350 and greater than 350 mm) using non-metric multidimensional scaling (NMDS) with two dimensions. NMDS is a method to visualize communities based on similarities to each other, with more similar communities grouping closer together. NMDS was conducted based on a fourth-root transformed Bray–Curtis dissimilarity matrix of the mean fish family abundance recorded in lionfish stomachs within each depth band and size category. The mean fish family abundance per stomach was used to control for differing numbers of lionfish stomachs examined and containing fish within each depth band and size category. Fourth-root transformation was used to reduce the relative influence of the most common prey fish families compared with rarer fish families. The matrix was constructed and NMDS fitted using the functions ‘vegdist’ and ‘metaMDS’ in the package ‘vegan’ [29] in R [30].

To identify whether differences in alert distance exist between heavily culled and unculled lionfish shallow-reef lionfish populations, we compared lionfish alert differences between Utila shallow culled (0–25 m), Utila MCE unculled (more than 40 m) and Tela shallow unculled (0–20 m) for individuals using an ANCOVA. Alert distance was natural log-transformed, and lionfish that did not appear to respond to the diver's presence until touched with the spear were given an alert distance of 1 cm. As previous studies on fish approach distances have consistently highlighted the importance of body size (see: [22]), we included lionfish total length in the model. Electronic supplementary material, 3–6 contain all raw data, and raw R code for analysis is contained in electronic supplementary material, 7.

## 3. Results

### 3.1. Lionfish distribution

**Figure 2.**
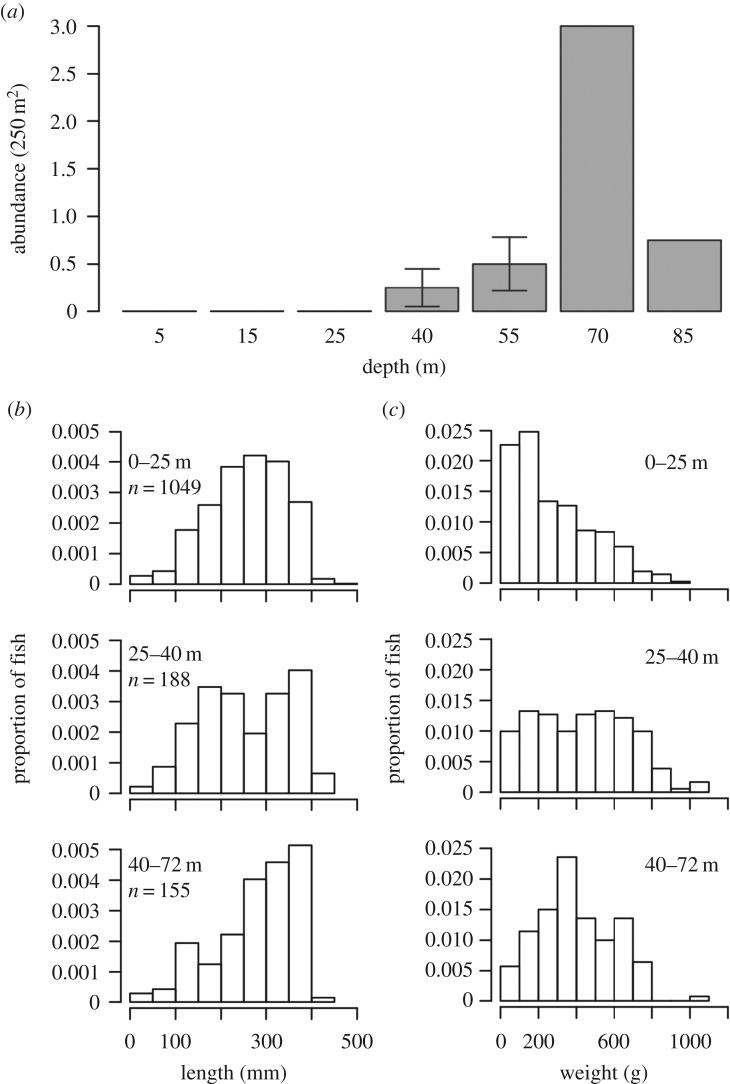
(*a*) Lionfish abundance, (*b*) body size and (*c*) weight changes. (*a*) Lionfish abundance recorded across the depth gradient. Lionfish abundance was recorded at all sites for 5, 15, 25 and 40 m (five sites), at Raggedy Cay, The Maze and Rocky Point for 55 m (three sites), and only at The Maze for 70 and 85 m (one site). Error bars indicate 95% CIs. No lionfish were recorded on any transects at 5, 15 or 25 m. (*b*) Lionfish length and (*c*) lionfish weight distributions by depth category based on speared lionfish measured and weighed post-dive. The total area of each histogram adds up to 1. The number (*n*) of lionfish collected within each depth category is indicated. See electronic supplementary material, 8 for Kolmogorov–Smirnov test results comparing lionfish length and weight distributions with depth.

When conducting lionfish density surveys across the depth gradient, no lionfish were found in Utila within the depth range of normal culling (5, 15 or 25 m) at any of the five sites surveyed (figure 2*a*). Lionfish were recorded at all depths deeper than where normal lionfish culling occurs (more than or equal to 40 m), with highest densities recorded at The Maze at 70 m, where the mean density was 3.0 individuals per 250 m^2^ (figure 2*a*). Our Utila lionfish dissection data incorporated lionfish collected from just below the surface (less than 1 m) through to the deepest lionfish collected at 72 m. Lionfish body length distributions were found to change with depth (figure 2*b*), with each depth band having a unique length distribution when compared with Kolmogorov–Smirnov tests (electronic supplementary material, 8). Utila lionfish mean body length was 25.8 ± 0.26 cm (mean ± s.e.) for shallow reefs (0–25 m) and 25.4 ± 0.7 cm for intermediate depths (25–40 m), with MCE lionfish (40–72 m) larger on average at 28.1 ± 0.7 cm. Similar to body length, lionfish weight increased with depth (figure 2*c*), with shallow lionfish weighing on average 0.28 ± 0.01 kg, compared with the intermediate (0.43 ± 0.02 kg) and MCE (0.40 ± 0.02 kg). While shallow lionfish had a unique weight distribution, there was no difference in weight distribution between intermediate and MCE depth lionfish (electronic supplementary material, 8).

**Figure 3.**
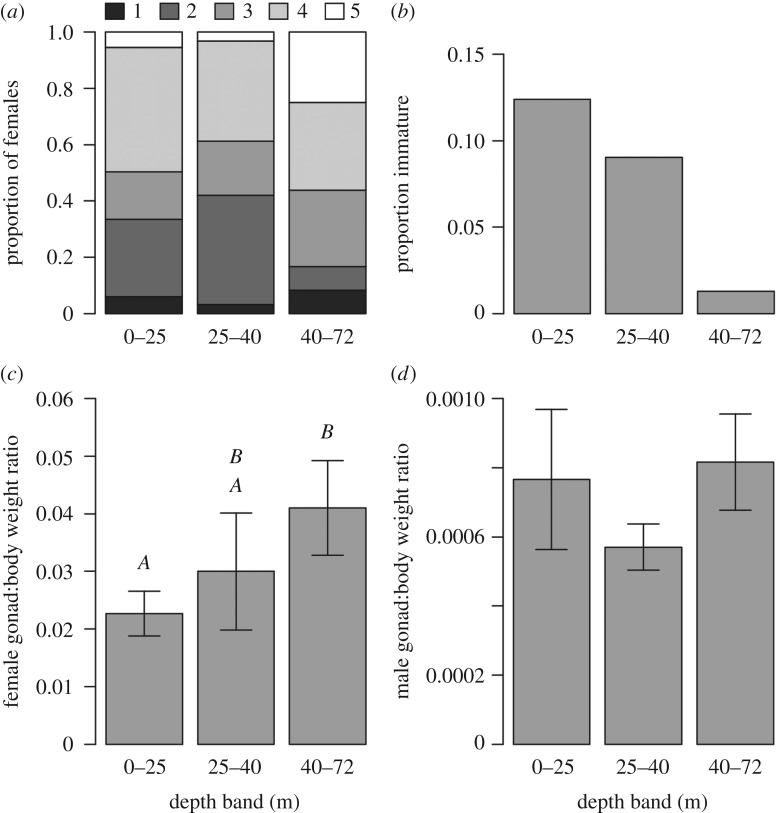
Lionfish maturity and gonad weight with depth. (*a*) Proportion of female lionfish with gonads at different levels of maturity. Female gonads were staged on a five-level score as follows: (1) immature, (2) early developing, (3) developing, (4) spawning capable and (5) actively spawning. Proportions calculated based on the following numbers of female lionfish per depth category: 365 (0–25 m), 31 (25–40 m) and 48 (40–72 m). (*b*) Number of immature lionfish speared in each depth band as a proportion of the total lionfish. Total lionfish numbers were: 1049 (0–25 m), 188 (25–40 m) and 155 (40–72 m). (*c*) Female gonad weight:body weight ratio by depth based on 117 (0–25 m), 29 (25–40 m) and 42 (40–72 m) lionfish. (*d*) Male gonad weight:body weight ratio by depth based on 141 (0–25 m), 71 (25–40 m) and 58 (40–72 m) lionfish. Error bars indicate 95% CIs. Letters on (*c*) designate significantly different groupings indicated by a post hoc Tukey honest significance difference test.

Female lionfish maturity changed with depth, both in terms of the proportion of individuals at different maturity stages (figure 3*a*) and the gonad:body weight ratio (figure 3*c*, *F*_2,185_ = 9.3, *p < *0.001). MCE female lionfish had greater mean gonad weight for their body weight than shallow females (figure 3*c*). While similar proportions of mature female lionfish (gonad stages 4 or 5) were recorded at shallow (49.6%), intermediate (38.7%) and MCE (56.3%) depths, substantially more females were actively spawning on MCEs, making up 25.0% of all MCE female lionfish (figure 3*a*). However, the male gonad:body weight ratio did not change with depth (figure 3*d*). Patterns in gonad stage and gonad:body weight ratio were probably influenced by the declining proportion of immature lionfish with increased depth (figure 3*b*). However, only 37% of immature lionfish could be sexed, with these sexed immature individuals representing 10% of all males and 5% of all females staged.

### 3.2. Lionfish condition and diet

**Figure 4.**
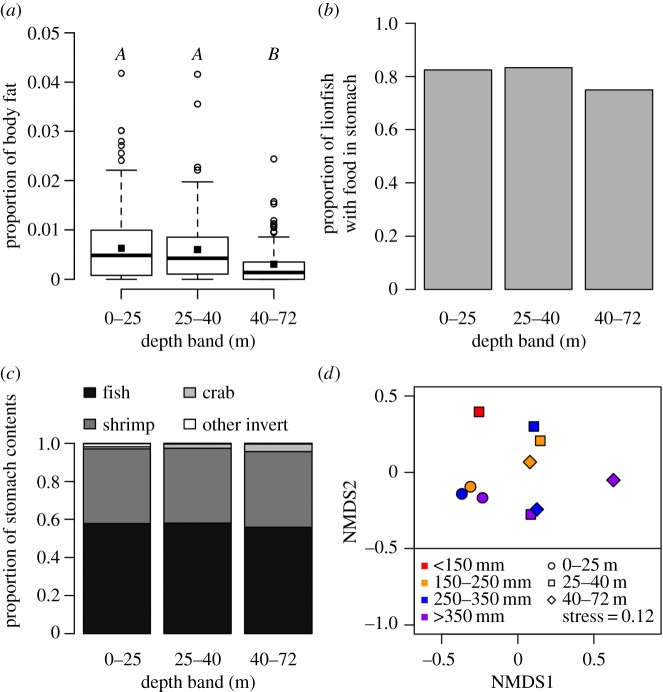
Lionfish condition and diet with depth. (*a*) Proportion of lionfish body weight composed of fat. The solid black line represents the median, with the box indicating the upper and lower quartiles, and whiskers representing the maximum or minimum observed value that is within 1.5 times the interquartile range of the upper or lower quartile, respectively. Open circles represent data points that fall outside the mean ± 1.5 times the interquartile range. Squares represent the mean. Letters designate significantly different groupings indicated by a post hoc Tukey honest significant difference test. (*b*) Proportion of lionfish with food in their stomachs. No difference was found between depth bands (electronic supplementary material, 9). (*c*) Proportion of lionfish stomach contents comprising fish, shrimp, crab and other invertebrates. (*d*) NMDS plot indicating similarities between fish family abundance found in lionfish stomachs belonging to different-sized lionfish at different depths. NMDS plot based on Bray–Curtis dissimilarities from fourth-root transformed mean fish family abundance per lionfish stomach in each depth band and length grouping. In total 392 lionfish stomachs contained fish identifiable to family level.

The proportion of body fat on lionfish changed significantly with depth (figure 4*a*, *F*_2,482_ = 8.9, *p < *0.001), with MCE lionfish having the lowest proportion of body fat at 0.0030 ± 0.0005, significantly lower than lionfish on intermediate depth reefs (0.0060 ± 0.0007, THSD: *p *= 0.005), or shallow reefs (0.0063 ± 0.0004, THSD: *p < *0.001). There was no difference in the proportion of body fat between lionfish on the shallow or intermediate depth reefs (figure 4*a*). Because we previously identified changes in lionfish body size with depth, to ensure that patterns in the proportion of body fat were not driven by changes in body size, we tested for a correlation between lionfish total length and proportion of body fat and found no relationship (Pearson's correlation coefficient: −0.01, *t* = −0.35, d.f. = 686, *p = *0.73). For female lionfish we tested for a relationship between gonad weight and the proportion of body fat while controlling for maturity level, fish weight and depth. We found that females with a lower proportion of body fat had increased gonad weight (electronic supplementary material, 9).

We found no difference in the proportion of lionfish stomachs containing food with depth, with 795 out of 964 inspected lionfish stomachs containing food items on shallow reefs, 110 out of 132 at intermediate depths and 81 out of 108 on MCEs (figure 4*b*; electronic supplementary material, 10). The diet composition of lionfish remained remarkably consistent across the depth gradient (figure 4*c*). Fish were the dominant component, accounting for between 56 and 58% of all stomach items across all depths. Shrimp were the second most common item, comprising 39–40% of all items at all depths. All other items combined accounted for less than 4% of lionfish diets, though crabs did show a slight increase in lionfish diets with depth, comprising 1.1% in the shallows, 2.2% at intermediate depths and 4.0% on MCEs. Algae of the genera *Halimeda* and *Dictyota* were found in the stomachs of seven shallow lionfish, but only accounted for 0.1% of the shallow lionfish stomach items overall. The NMDS plot of fish family abundance found in lionfish stomachs suggests that the shallows have a unique diet assemblage (figure 4*d*), with the intermediate and MCE depth assemblages placed closer to one another than to that of the shallows. Gobiidae were the most consumed fish family in the shallows and on MCEs, making up 31% and 43% of fish found in lionfish stomachs, respectively (table 1). Both Pomacentridae and Scaridae made up a large proportion of fish consumed on MCEs by lionfish, yet made up a much smaller proportion in the shallows (table 1).

**Table 1.** Proportion of fish by family recorded within all lionfish stomachs within each depth band. Each column adds up to 1.0, and is based on grouping all fish identifiable to family level from all lionfish stomachs within a depth band. Results based on 392 lionfish containing fish identifiable to family level within their stomachs, with 351 from 0–25 m, 27 from 25–40 m and 14 from 40–72 m.

	proportion of fish within depth band		
family	0–25 m	25–40 m	40–72 m
Acanthuridae	0.02	0.00	0.00
Apogonidae	0.00	0.09	0.00
Balistidae	0.01	0.00	0.00
Blenniidae	0.26	0.09	0.07
Gobiidae	0.31	0.13	0.43
Haemulidae	0.01	0.04	0.00
Holocentridae	0.01	0.00	0.00
Labridae	0.17	0.09	0.07
Monacanthidae	0.03	0.17	0.07
Pomacentridae	0.05	0.26	0.14
Scaridae	0.08	0.04	0.21
Serranidae	0.03	0.09	0.00
Synodontidae	0.01	0.00	0.00
Tetraodontidae	0.01	0.00	0.00

### 3.3. Lionfish behaviour

We identified larger alert distances for lionfish on shallow culled reefs around Utila (median = 25 cm) than shallow reefs without culling in Tela (median = 5 cm), representing lionfish allowing divers on average 400% closer on shallow Telan reefs (table 2). Yet lionfish alert distances on Utilan MCEs (median = 25 cm) were no different from those on shallow Utilan reefs (table 2; THSD: *p *= 0.98). Our alert distance data were based on 372 shallow Telan, 30 shallow Utilan and 22 MCE Utilan lionfish. Larger lionfish had greater alert distances than smaller individuals (table 2). For example, from our model we would predict that 10 cm and 30 cm total length lionfish would on shallow Utilan reefs have alert distances of 20.0 cm and 97.7 cm, respectively.

**Table 2.** ANCOVA results testing differences in lionfish alert distance with depth in Utila and Tela. Alert distance was natural log-transformed. The intercept represents the alert distance for Tela, with the Utila shallow culled and Utila MCE unculled estimates given as the difference from the intercept, and lionfish total length estimate given as a slope, indicating that as lionfish length increases, alert distance increases. Alert distance data based on 372 shallow Telan, 30 shallow Utilan and 22 MCE Utilan lionfish.

	estimate	s. e.	*t*-value	*p*-value
intercept (Tela unculled shallow)	1.79	0.16	10.93	<0.001
Utila shallow culled	1.30	0.19	6.90	<0.001
Utila MCE unculled	1.35	0.21	6.30	<0.001
lionfish total length	0.01	0.01	2.09	0.037

## 4. Discussion

We identified that MCEs surrounding Utila contain greater densities of lionfish than shallow reefs, suggesting that MCEs act as a refuge for invasive lionfish from the current depth range of culling in Utila. Lionfish populations on deeper reefs comprised larger, heavier individuals, with higher proportions of actively spawning female lionfish that had greater gonad weight for their body size than lionfish found on shallower reefs. This suggests that this MCE population refuge not only allows individual lionfish to evade culling, but is also a preferred habitat of mature fish. In addition, we identified changes in lionfish alert distance between reefs with culling and those without, but crucially no difference between culled shallow reefs and adjacent unculled MCEs, suggesting that MCE lionfish have been conditioned to be wary of divers. Taken together, our findings suggest depth-dependent effects of culling, but also provide some support to lionfish undergoing ontogenetic migrations from shallow reefs to MCEs, highlighting the importance of considering MCE lionfish populations in lionfish management decision-making.

### 4.1. Greater lionfish density on mesophotic coral ecosystems than on shallow reefs

We found greater lionfish densities on MCEs than on shallow reefs, with no lionfish recorded on transects at 5, 15 or 25 m. Considering the high shallow-reef lionfish densities reported from many western Atlantic locations without culling (see [4,17]) including from shallow unculled sites in Honduras [31], combined with the previous reported declines in shallow-reef lionfish abundance when culled [8,23], our results suggest that the Utila lionfish culling is reducing shallow lionfish numbers. Our results also indicate, however, that reefs below the range of lionfish removal retain substantial lionfish populations.

Finer patterns in lionfish density between individual transect depths should be treated with caution. Within MCEs (40–85 m depth) fewer sites were surveyed at 55 m, and only one site at 70 and 85 m. The high lionfish densities at 70 m compared with 85 m at the Maze were probably influenced by reef habitat availability, with MCEs at this site characterized by steep walls broken by ledges approximately 20 m wide [26]. Our 70 m transects at the Maze coincided with one such ledge, providing a large area of reef habitat, while our 85 m transects were on a wall. While in this study we were restricted to video transects because of limited dive survey time on MCEs [26], studies on shallow reefs have cautioned that transects may underestimate lionfish density, with biases dependent on habitat structure and lionfish body size [32]. However, our results appear robust when compared with other lionfish surveys of Utila, and patterns of reef complexity. For example, Henly [31] surveyed shallow-reef lionfish density in Utila in 2015 using targeted lionfish searches. Despite including extensive visual searches of reef crevices, they recorded low lionfish densities at 5 and 10 m depth in Utila at approximately 0.16 individuals per 250 m^2^, and no individuals at a 15 m depth. While we did not record any lionfish on transects at 5, 15 or 25 m depth, the 5 and 10 m density reported by Henly [31] is far lower than the densities we recorded with video transects from MCEs in Utila (up to 3.0 individuals per 250 m^2^). This implies that regardless of whether some lionfish were missed by survey limitations in our shallow transects, the high numbers recorded on MCEs indicate that lionfish densities on MCEs are still substantially greater than those in the shallows. In addition, a previous study of benthic habitats around Utila found little difference in benthic communities between the 25 and 40 m depths [25]. This suggests that the absence of lionfish on our 25 m depth transects compared to those conducted at 40 m probably reflects a genuine difference in lionfish density. Therefore, our lionfish densities from transects appear to fit with previous reported patterns from extensive lionfish visual surveys, and changing patterns of complexity. Regardless of variation in lionfish density between specific transect depths, our results clearly indicate the refuge role played by MCEs for lionfish in the presence of shallow culling.

### 4.2. Depth-dependent lionfish population attributes across shallow reef to mesophotic coral ecosystem depth gradients

Patterns in our lionfish dissection results could partially be explained by previous suggestions of lionfish ontogenetic migrations extending onto MCEs [15,20], or may be driven primarily by depth-specific effects of culling, which is known to alter lionfish populations [8]. We detected declining proportions of immature lionfish with depth, supporting previous observations of ontogenetic movements in lionfish [20]. In addition to our results agreeing with previously documented increases in lionfish body size with depth [15,21], we also found a greater proportion of actively spawning female lionfish on MCEs, and gonad weight increased as a proportion of overall body weight for female lionfish at increased depth (figure 3). Yet we found no relationship between male gonad:body weight ratio and depth. This may have been affected by difficulties in sexing immature lionfish, which were more common at shallow depths. Had it been possible to sex all immature lionfish, it is possible that we may have observed an increase in male gonad:body weight ratio with increased depth. However, the proportion of sexually mature female lionfish (combining those capable of spawning and those actively spawning) appeared consistent across the depth gradient, supporting the idea that reproductively mature males would be expected to be found across all depths. Detecting differences in female lionfish maturity across depths may also suffer from under-inclusion of immature females; however, as maturity was assessed on a scale of 1–5, and mature female gonads reach greater weight than males, differences with depth were still apparent.

We analysed lionfish stomachs and found no difference in the proportion of lionfish stomachs containing food with depth. Despite this, there were differences in the proportion of body fat with depth, with MCE lionfish having lower body fat levels than shallow-reef lionfish. We found that female lionfish with lower body fat levels have greater gonad weights while controlling for lionfish body size and maturity. This suggests that lower fat stores could be indicative of energy being partitioned to gonad development and reproductive output, with the most reproductively active lionfish found on MCEs.

Overall, our results suggest that individual lionfish on MCEs are larger and in the case of females have larger gonads for their size than those in the shallows. It is not clear which mechanism drives these patterns. For example, culling has been found to shift lionfish length distributions towards smaller fish [8], therefore our observed pattern in length/weight distribution could be generated by shallow-reef culling. The observed pattern also fits with previous studies documenting increased body size and maturity at greater depths (ontogenetic migration) across shallow reef to MCE gradients for native unculled western Atlantic reef fish [33,34]. It is well established in many fish species that larger older females have greater fecundity than smaller younger females, and that larger older females produce larvae with greater survival rates than younger females [35,36]. If the observed pattern is caused by an ontogenetic migration, this suggests that in locations such as Utila, where not only are there substantial MCE lionfish populations despite shallow-reef culling, but also these individuals are larger than their shallow-reef conspecifics, MCE lionfish may be disproportionately responsible for continued new shallow-reef lionfish recruitment.

Experiments conducted in The Bahamas have shown that regular culling increases alert distances in invasive lionfish [23]. Our results agree with this finding, as we observed greater alert distances for shallow-reef lionfish in Utila (culled) than in Tela (unculled). However, we also found (i) no change in lionfish alert distance between shallow reefs (culled) and MCEs (unculled) in Utila and (ii) significantly increased alert distances on both culled Utilan shallow reefs and unculled Utilan MCEs compared with unculled Telan shallow reefs. With lionfish culling around Utila limited to shallow reefs (less than 30 m), we might have expected to see lower alert distances in MCE lionfish populations, similar to those observed on the reefs of Tela. However, our results fit in the context of lionfish ontogenetic movements if individuals surveyed on MCEs have previously lived within the culling depth range prior to migrating onto MCEs as they mature. This suggests that behavioural responses to predator recognition are likely to be retained when lionfish move beyond the range of culling, which agrees with studies of other reef fish species on spearfished sites, which are likely to flee in response to future encounters with divers regardless of whether they are spearfishers or not [22]. Increased alert distances with increased body length, such as that observed in lionfish, has been widely observed in other fish species subjected to regular spearfishing, though it is not clear what drives these patterns [22]. Lionfish alert distances may also be affected by factors such as reef complexity, as in many cases lionfish fled into reef crevices when approached. Shallow-reef habitat complexity is greater in Tela Bay than in Utila [24], and habitat complexity further declines with increased depth in Utila [25]. If alert distance was correlated with habitat complexity (the more available shelter for lionfish to hide, the closer they allow divers to approach), we would expect shallow Telan reefs to have the lowest alert distance, followed by shallow Utilan reefs, with Utilan MCEs having the greatest alert distances. Yet in our results we do not see this pattern, though we recommend that future studies should specifically assess the complexity of habitats that lionfish are associated with when considering behavioural questions.

While increases in the mean length, weight and female gonad:body weight ratio, combined with reduced numbers of immature fish at increased depth are used as indicators of ontogenetic migrations in other western Atlantic reef fish species [34,37], they do not conclusively indicate an ontogenetic migration occurring in lionfish. We found mature females at all depths around Utila, and no change in the male gonad:body weight ratio with depth, both of which are inconsistent with ontogenetic migrations. Previous shallow-reef culling has been found to result in decreased mean lionfish body length in shallow lionfish populations [8], possibly because of bias by divers to remove larger, more easily detectable lionfish [32]. Therefore, while many of our dissection results are consistent with the lionfish depth patterns expected from ontogenetic migrations, these results could be explained by the presence of culling. However, the consistency of lionfish alert distances across the depth gradient in Utila supports the idea that these patterns were generated by an ontogenetic migration, and are not just an artefact of removing the most mature individuals from the shallows. To further test this hypothesis, we recommend that future studies compare age estimates from lionfish otoliths across the depth gradient between locations with and without culling. Regardless of the mechanism causing the observed differences in lionfish size and maturity across the depth gradient, our results still highlight that MCEs are acting as depth refuges for lionfish in Utila, and that these MCE lionfish may be disproportionately responsible for new lionfish recruits entering the population.

### 4.3. Differences in lionfish diet across the depth gradient

We analysed lionfish stomach contents across the depth gradient and found that fish were the dominant diet component for all depths. While this is consistent with shallow-reef findings across the invaded and native lionfish range [38–40], some studies have suggested that lionfish diets are mostly determined by the local abundance of fish and invertebrate species [39], while others have suggested selectivity in lionfish diets based on prey traits [41]. Previous studies on Utila have identified declines in native fish abundance and biomass with increased depth [25], and that large planktonic reef-associated invertebrate (more than 2 mm body size) abundance increases with depth [42]. We therefore could have expected to see a shift to increased invertebrate composition of lionfish diets on MCEs. However, when considering fish families consumed by lionfish, we identified that Gobiidae comprised 43% of fish in MCE lionfish diets. As Gobiidae are small benthic-associated cryptic fish, it is likely that as previous Utila fish surveys across the depth gradient have used diver-operated stereo-video system transects [25], they have under-recorded Gobiidae abundance on reefs, as this technique is known to under-report small fish on Utilan MCEs [33]. A study from Curaçao identified the greatest numbers of Gobiidae recruits in the 20–30 m range when compared with 10 m and 40 m [43]. Yet we found Gobiidae made up the lowest proportion of fish in lionfish stomachs in the 25–40 m range, with greater proportions in the 0–25 m and 40–72 m depth bands. Our NMDS plot (figure 4*d*) shows that depth, rather than lionfish body size plays a major role in determining lionfish diets, and suggests that lionfish diets at the intermediate depths (25–40 m) and on MCEs (40–72 m) are more similar than shallow diets. This pattern fits with recorded fish community changeover in prey items across the depth gradient [25,33]. Previous work has suggested that lionfish culling can lead to a decrease in the number of stomach items, and in some cases can lead to a shift in diet from fish to invertebrates as lionfish change their hunting behaviour [8]. Despite culling being restricted to shallow reefs, we found no evidence of reduced feeding at shallow depths, or a greater invertebrate contribution to lionfish diets in the shallows.

### 4.4. Management approaches for mesophotic coral ecosystem lionfish

In most western Atlantic countries affected by the lionfish invasion, the most commonly used approach for lionfish population management has been culling by divers using hand spears [44]. Culling has been found to be effective in reducing lionfish abundance on shallow reefs [8], which in turn can help shallow native reef fish communities recover [45]. Culling is normally managed by governments, but typically carried out by volunteer recreational divers, limiting removal efforts in many locations to less than 30 m. Technical diving requirements have meant lionfish removal on many western Atlantic MCEs has not been possible. However, within the dive community there is increased interest in technical diving [46], leading some lionfish management organizations to specifically engage technical divers in community-based lionfish management activities. For example, in Utila, during the July 2016 annual lionfish derby, the Bay Islands Conservation Association included a technical diving category for the first time [15]. Public lionfish derbies are a widely used management tool to increase lionfish removal from reefs while simultaneously increasing public engagement and awareness of marine conservation, with teams competing for prizes based on the number and sizes of lionfish collected [47]. By incorporating a technical diving category, and specifically encouraging dive centres with technical diving capabilities to compete, it was possible to expand lionfish removal efforts onto MCEs.

Other methods have been proposed for lionfish removal from MCEs that may be more appropriate for areas with limited technical diving, including trapping [48] and more novel techniques such as underwater robotics [49]. Lionfish traps have shown promise in some areas, for example, in Bermuda over 1200 lionfish were removed between September 2013 and March 2014 in traps from a 40–80 m depth [50]. While these lionfish represented by-catch in commercial lobster traps, it has shown that substantial numbers of lionfish can be removed by trapping. This has driven interest in developing lionfish-specific traps, minimizing by-catch of other species, which can be more widely used for MCE lionfish removal [50]. While underwater robots for removal of invasive lionfish are not yet readily available and still require further development, several trials are underway using robots to stun lionfish with an electric shock allowing their removal [49]. Recently, robots using a vision-based identification and tracking system have been tested to aid population control of crown-of-thorn starfish on the Great Barrier Reef [51,52]. This system allows an underwater robot to identify a crown-of-thorn starfish against a reef backdrop, follow its movement, and then administer a lethal injection [49,51]. Similar technological advances could play a crucial role in future lionfish removal efforts, especially from habitats beyond recreational diving limits such as MCEs.

## 5. Conclusion

We studied invasive lionfish populations across the shallow reef to MCE depth gradient, identifying a high density of lionfish below the maximum depth of most culling. These deeper reef lionfish were found to be larger, with females having heavier gonad weights for their body weight and a greater proportion of the population actively spawning compared to those found on shallow reefs. This raises the possibility that deep reef lionfish may be undermining current shallow-reef-focused culling efforts. In addition, we identified that MCE lionfish display behavioural responses to divers consistent with living on a culled reef, despite being below the range of culling, supporting the idea that lionfish ontogenetic migrations may extend onto MCEs. Our results highlight the need for lionfish management plans to consider the importance of MCEs to lionfish life cycles, thereby improving the effectiveness of culling programmes.

## Supplementary Material

ESM 1

## Supplementary Material

ESM 2

## Supplementary Material

ESM 3

## Supplementary Material

ESM 4

## Supplementary Material

ESM 5

## Supplementary Material

ESM 6

## Supplementary Material

ESM 7

## Supplementary Material

ESM 8

## Supplementary Material

ESM 9

## Supplementary Material

ESM 10

## Data accessibility

Electronic supplementary material, 3–6 contain all raw data, and raw R code for analysis is contained in electronic supplementary material, 7.

## References

[1] Schofield PJ 2009 Geographic extent and chronology of the invasion of non-native lionfish (*Pterois volitans* [Linnaeus 1758] and *P. miles* [Bennett 1828]) in the Western North Atlantic and Caribbean Sea. Aquat. Invasions 4, 473–479. (doi:10.3391/ai.2009.4.3.5)

[2] Schofield PJ 2010 Update on geographic spread of invasive lionfishes (*Pterois volitans* [Linnaeus, 1758] and *P. miles* [Bennett, 1828]) in the Western North Atlantic Ocean, Caribbean Sea and Gulf of Mexico. Aquat. Invasions 5, S117–S122. (doi:10.3391/ai.2010.5.S1.024)

[3] Freshwater DW et al. 2009 Mitochondrial control region sequence analyses indicate dispersal from the US East Coast as the source of the invasive Indo-Pacific lionfish *Pterois volitans* in the Bahamas. Mar. Biol. 156, 1213–1221. (doi:10.1007/s00227-009-1163-8)

[4] Darling ES, Green SJ, O'Leary JK, Côté IM 2011 Indo-Pacific lionfish are larger and more abundant on invaded reefs: a comparison of Kenyan and Bahamian lionfish populations. Biol. Invasions 13, 2045–2051. (doi:10.1007/s10530-011-0020-0)

[5] Albins MA, Hixon MA 2008 Invasive Indo-Pacific lionfish *Pterois volitans* reduce recruitment of Atlantic coral-reef fishes. Mar. Ecol. Progr. Ser. 367, 233–238. (doi:10.3354/meps07620)

[6] Green SJ, Akins JL, Maljković A, Côté IM 2012 Invasive lionfish drive Atlantic coral reef fish declines. PLoS ONE 7, e32596 (doi:10.1371/journal.pone.0032596) 22412895 10.1371/journal.pone.0032596 PMC3296711

[7] Morris JA 2012 Invasive lionfish: a guide to control and management. Marathon, FL: Gulf and Caribbean Fisheries Institute Special Publication Series Number 1.

[8] Frazer TK, Jacoby CA, Edwards MA, Barry SC, Manfrino CM 2012 Coping with the lionfish invasion: can targeted removals yield beneficial effects? Rev. Fish Sci. 20, 185–191. (doi:10.1080/10641262.2012.700655)

[9] Arias-González JE, González-Gándara C, Luis Cabrera J, Christensen V 2011 Predicted impact of the invasive lionfish *Pterois volitans* on the food web of a Caribbean coral reef. Environ. Res. 111, 917–925. (doi:10.1016/j.envres.2011.07.008) 21840517 10.1016/j.envres.2011.07.008

[10] Menza C, Kendall M, Hile S 2008 The deeper we go the less we know. Rev. Biol. Trop. 56, 11–24.

[11] Baker EK, Puglise KA, Harris PT 2016 Mesophotic coral ecosystems—a lifeboat for coral reefs? Nairobi, Kenya: The United Nations Environment Programme and GRID-Arendal.

[12] Andradi-Brown D et al. 2016 Threats to mesophotic coral ecosystems and management options. In Mesophotic coral ecosystems—a lifeboat for coral reefs? (eds Baker EK, Puglise KA, Harris PT), pp. 67–82. Nairobi, Kenya: The United Nations Environment Programme and GRID-Arendal.

[13] Lesser MP, Slattery M 2011 Phase shift to algal dominated communities at mesophotic depths associated with lionfish (*Pterois volitans*) invasion on a Bahamian coral reef. Biol. Invasions 13, 1855–1868. (doi:10.1007/s10530-011-0005-z)

[14] Nuttall MF, Johnston MA, Eckert RJ, Embesi JA, Hickerson EL, Schmahl GP 2014 Lionfish (*Pterois volitans* [Linnaeus, 1758] and *P. miles* [Bennett, 1828]) records within mesophotic depth ranges on natural banks in the Northwestern Gulf of Mexico. BioInvasions Records 3, 111–115. (doi:10.3391/bir.2014.3.2.09)

[15] Andradi-Brown DA et al 2017 Large-scale invasion of Western Atlantic mesophotic reefs by invasive lionfish potentially undermines culling-based management. Biol. Invasions 19, 939–954. (doi:10.1007/s10530-016-1358-0)

[16] Brokovich E, Einbinder S, Shashar N, Kiflawi M, Kark S 2008 Descending to the twilight-zone: changes in coral reef fish assemblages along a depth gradient down to 65 m. Mar. Ecol. Progr. Ser. 371, 253–262. (doi:10.3354/meps07591)

[17] Kulbicki M et al. 2012 Distributions of Indo-Pacific lionfishes *Pterois* spp. in their native ranges: implications for the Atlantic invasion. Mar. Ecol. Progr. Series 446, 189–205. (doi:10.3354/meps09442)

[18] Wright DJ 2005 Report of HURL cruise KOK0510: submersible dives and multibeam mapping to investigate benthic habitats of Tutuila, American Samoa. Oregon State University Scholars Archive. (http://hdl.handle.net/1957/4282)

[19] Slattery M, Lesser MP 2014 Allelopathy in the tropical alga *Lobophora variegata* (Phaeophyceae): mechanistic basis for a phase shift on mesophotic coral reefs? J. Phycol. 50, 493–505. (doi:10.1111/jpy.12160) 26988322 10.1111/jpy.12160

[20] Claydon J, Calosso MC, Traiger SB 2012 Progression of invasive lionfish in seagrass, mangrove and reef habitats. Mar. Ecol. Progr. Ser. 448, 119–129. (doi:10.3354/meps09534)

[21] de León R, Vane K, Bertuol P, Chamberland VC, Simal F, Imms E, Vermeij M 2013 Effectiveness of lionfish removal efforts in the southern Caribbean. Endang. Species Res. 22, 175–182. (doi:10.3354/esr00542)

[22] Januchowski-Hartley FA, Graham NAJ, Feary DA, Morove T, Cinner JE 2011 Fear of fishers: human predation explains behavioral changes in coral reef fishes. PLoS ONE 6, e22761 (doi:10.1371/journal.pone.0022761) 21853046 10.1371/journal.pone.0022761 PMC3154266

[23] Côté IM, Darling ES, Malpica-Cruz L, Smith NS, Green SJ, Curtis-Quick J, Layman C 2014 What doesn't kill you makes you wary? Effect of repeated culling on the behaviour of an invasive predator. PLoS ONE 9, e94248 (doi:10.1371/journal.pone.0094248) 24705447 10.1371/journal.pone.0094248 PMC3976393

[24] Bodmer MDV, Rogers AD, Speight MR, Lubbock N, Exton DA 2015 Using an isolated population boom to explore barriers to recovery in the keystone Caribbean coral reef herbivore *Diadema antillarum*. Coral Reefs 34, 1011–1021. (doi:10.1007/s00338-015-1329-4)

[25] Andradi-Brown DA, Gress E, Wright G, Exton DA, Rogers AD 2016 Reef fish community biomass and trophic structure changes across shallow to upper-mesophotic reefs in the Mesoamerican Barrier Reef, Caribbean. PLoS ONE 11, e0156641 (doi:10.1371/journal.pone.0156641) 27332811 10.1371/journal.pone.0156641 PMC4917088

[26] Andradi-Brown DA, East A, Shepherd LM, Stockdale EJ, Rogers AD 2016 Challenges and opportunities in conducting mesophotic reef research. Reef Encounter 31, 26–31.

[27] Green SJ, Akins JL, Morris JA Jr 2012 Lionfish Dissection: Techniques and applications. NOAA Technical Memorandum NOS NCCOS 139, 24 p.

[28] Benjamini Y, Yekutieli D 2001 The control of the false discovery rate in multiple testing under dependency. Ann. Stat. 29, 1165–1188. (doi:10.1214/aos/1013699998)

[29] Oksanen J, Kindt R, Legendre P, O'Hara B, Simpson GL, Solymos P, Stevens MHH, Wagner H 2013 vegan: community ecology package. *cran.r-project.org*.

[30] R Core Team. 2013 R: A language and environment for statistical computing. *R-project.org*.

[31] Henly L 2017 Impacts of culling invasive lionfish (*Pterois* spp.) on native reef fish assemblages in Honduras. The Plymouth Student Scientist 10, 22–40.

[32] Green SJ, Tamburello N, Miller SE, Akins JL, Côté IM 2013 Habitat complexity and fish size affect the detection of Indo-Pacific lionfish on invaded coral reefs. Coral Reefs 32, 413–421. (doi:10.1007/s00338-012-0987-8)

[33] Andradi-Brown DA, Macaya-Solis C, Exton DA, Gress E, Wright G, Rogers AD 2016 Assessing Caribbean shallow and mesophotic reef fish communities using baited-remote underwater video (BRUV) and diver-operated video (DOV) survey techniques. PLoS ONE 11, e0168235 (doi:10.1371/journal.pone.0168235) 27959907 10.1371/journal.pone.0168235 PMC5154558

[34] Goldstein ED, D'Alessandro EK, Sponaugle S 2016 Demographic and reproductive plasticity across the depth distribution of a coral reef fish. Sci. Rep. 6, 34077 (doi:10.1038/srep34077) 27677948 10.1038/srep34077 PMC5039716

[35] Birkeland C, Dayton P 2005 The importance in fishery management of leaving the big ones. Trends Ecol. Evol. 20, 356–358. (doi:10.1016/j.tree.2005.03.015) 16701393 10.1016/j.tree.2005.03.015

[36] Hixon MA, Johnson DW, Sogard SM 2014 BOFFFFs: on the importance of conserving old-growth age structure in fishery populations. ICES J. Mar. Sci. 71, 2171–2185. (doi:10.1093/icesjms/fst200)

[37] de la Morinière E Cocheret, Pollux BJA, Nagelkerken I, van der Velde G 2002 Post-settlement life cycle migration patterns and habitat preference of coral reef fish that use seagrass and mangrove habitats as nurseries. Estuarine, Coastal and Shelf Sci. 55, 309–321. (doi:10.1006/ecss.2001.0907)

[38] Green SJ, Akins JL, Côté IM 2011 Foraging behaviour and prey consumption in the Indo-Pacific lionfish on Bahamian coral reefs. Mar. Ecol. Progr. Ser. 433, 159–167. (doi:10.3354/meps09208)

[39] Muñoz RC, Currin CA, Whitfield PE 2011 Diet of invasive lionfish on hard bottom reefs of the Southeast USA: insights from stomach contents and stable isotopes. Mar. Ecol. Progr. Ser. 432, 181–193. (doi:10.3354/meps09154)

[40] Cure K, Benkwitt CE, Kindinger RL, Pickering EA, Pusack TJ, McIlwain JL, Hixon MA 2012 Comparative behavior of red lionfish *Pterois volitans* on native Pacific versus invaded Atlantic coral reefs. Mar. Ecol. Progr. Ser. 467, 181–192. (doi:10.3354/meps09942)

[41] Green SJ, Côté IM 2014 Trait-based diet selection: prey behaviour and morphology predict vulnerability to predation in reef fish communities. J. Anim. Ecol. 83, 1451–1460. (doi:10.1111/1365-2656.12250) 24861366 10.1111/1365-2656.12250

[42] Andradi-Brown DA, Head CEI, Exton DA, Hunt CL, Hendrix A, Gress E, Rogers AD 2017 Identifying zooplankton community changes between shallow and upper-mesophotic reefs on the Mesoamerican Barrier Reef, Caribbean. PeerJ 5, e2853 (doi:10.7717/peerj.2853) 28168098 10.7717/peerj.2853 PMC5289443

[43] Luckhurst BE, Luckhurst K 1977 Recruitment patterns of coral reef fishes on the fringing reef of Curaçao, Netherlands Antilles. Can. J. Zool. 55, 681–689. (doi:10.1139/z77-089)

[44] Côté IM, Akins L, Underwood E, Curtis-Quick J, Green SJ 2014 Setting the record straight on invasive lionfish control: culling works. PeerJ PrePrints 2, e398v1 (doi:10.7287/peerj.preprints.398v1)

[45] Green SJ, Dulvy NK, Brooks AML, Akins JL, Cooper AB, Miller S, Côté IM 2014 Linking removal targets to the ecological effects of invaders: a predictive model and field test. Ecol. Appl. 24, 1311–1322. (doi:10.1890/13-0979.1) 10.1890/13-0979.1 29160656

[46] Mitchell SJ, Doolette DJ 2013 Recreational technical diving part 1: an introduction to technical diving methods and activities. Diving and Hyperbaric Med. 43, 86–93. 23813462

[47] Malpica-Cruz L, Chaves LCT, Côté IM 2016 Managing marine invasive species through public participation: Lionfish derbies as a case study. Mar. Policy 74, 158–164. (doi:10.1016/j.marpol.2016.09.027)

[48] Bogdanoff AK, Akins JL, Morris JA, 2013 GCFI Lionfish Workgroup. 2014 Invasive lionfish in the marketplace: challenges and opportunities. In Proc. 66th Gulf and Caribbean Fisheries Institute, November 4–8, 2013 Corpus Christi, TX, pp. 140–147.

[49] Sutherland WJ et al. 2017 A 2017 horizon scan of emerging issues for global conservation and biological diversity. Trends Ecol. Evol. 32, 31–40. (doi:10.1016/j.tree.2016.11.005) 27955953 10.1016/j.tree.2016.11.005

[50] Pitt JM, Trott TM 2015 Trapping lionfish in Bermuda, Part II: Lessons learned to date. In Proc. 67th Gulf and Caribbean Fisheries Institute, November 3–7 2014, Christ Church, Barbados, pp. 221–224.

[51] Dayoub F, Dunbabin M, Corke P 2015 Robotic detection and tracking of crown-of-thorns starfish. In IEEE/RSJ Int. Conf. Intelligent Robots and Systems (IROS), Hamburg, Germany, 28 September–2 October, pp. 1921–1928. (doi:10.1109/IROS.2015.7353629)

[52] Platt JR 2015 The starfish assassin. Sci. Am. 314, 16 (doi:10.1038/scientificamerican0116-16) 10.1038/scientificamerican0116-16 26887184

