# Supplementary material for: Depth-dependent effects of culling—do mesophotic lionfish populations undermine current management?
Source: R Soc Open Sci. 2017 May 24;4(5):170027. doi: 10.1098/rsos.170027 (PMC5451808; doi:10.1098/rsos.170027)
Supplement: ESM 1 [file rsos170027supp1.docx]

**ESM 1. GPS locations of lionfish abundance survey locations around Utila, Honduras.** All GPS coordinates are given in WGS 84. GPS locations indicate the center point of the transects, with divers descending on the reef wall at these point to the respective survey depth. Once at the survey depth, survey teams set up the stereo-video cameras system and then swam 10 m along the reef prior to starting the transect recording lionfish abundance. Following the first transect completion, a 10 m interval was left and then a second transect was filmed. Two transects were conducted in each direction along the reef wall from the GPS coordinates stated.

| Site Name | Latitude | Longitude |
| --- | --- | --- |
| Raggedy Cay (West End) | 16.09065964 | -86.99410150 |
| The Maze | 16.11266214 | -86.94911793 |
| Little Bight | 16.07926302 | -86.92942222 |
| Coral View | 16.08823274 | -86.91094506 |
| Rocky Point | 16.08784039 | -86.88423403 |
