# Supplementary material for: Depth-dependent effects of culling—do mesophotic lionfish populations undermine current management?
Source: R Soc Open Sci. 2017 May 24;4(5):170027. doi: 10.1098/rsos.170027 (PMC5451808; doi:10.1098/rsos.170027)
Supplement: ESM 2 [file rsos170027supp2.docx]

**ESM 2**

Utila lionfish dissection data came from four sources: (i) Bay Islands Conservation Association (BICA) lionfish derbies, (ii) Utila Whale Shark and Oceanic Research Center lionfish culling program, (iii) Operation Wallacea lionfish monitoring program and (iv) the University of Oxford Thinking Deep MCE expedition. The contributions to this study from each source are shown in ESM2 Table 1, with lionfish collected between 2014–2016.

ESM 2 Table 1. Source of Utila lionfish used in the study including collection date and number of lionfish.

| Source | Month(s) | Year | Number of lionfish |
| --- | --- | --- | --- |
| Bay Islands Conservation Association Lionfish Derby | Jul | 2015 | 118 |
|  | Jul | 2016 | 195 |
| Whale Shark and Oceanic Research Center Lionfish Culling Program | Apr - Dec | 2015 | 526 |
|  | Jan - Aug | 2016 | 365 |
| Operation Wallacea Lionfish Monitoring | Jun - Aug | 2014 | 60 |
|  | Jun - Aug | 2015 | 44 |
|  | Jun - Aug | 2016 | 53 |
| University of Oxford Thinking Deep Expedition | Aug - Sep | 2015 | 31 |

Lionfish derbies are a community approach to lionfish removal in the western Atlantic, with divers forming teams to compete against each other to remove the most lionfish from the reefs. Dive teams collecting lionfish in the BICA lionfish derbies were briefed on the importance of collecting all lionfish regardless of size. To minimize risk of biases in size of fish collected, the derby awarded prizes for the team with the most fish collected, and to the team collecting the largest, smallest and median sized fish. The depth of collection was recorded for all lionfish within three depth intervals; shallow: 0–25 m, intermediate: 25–40 m and MCE: >40 m. Lionfish collected from sources other than the derby were collected on a mixture of dedicated lionfish spearing dives and opportunistically on other survey and recreational dives during the time period indicated in Table, with exact depth of collection recorded, or in the case of many fish being collected, using the same depth bands as the derby.
