# Supplementary material for: Depth-dependent effects of culling—do mesophotic lionfish populations undermine current management?
Source: R Soc Open Sci. 2017 May 24;4(5):170027. doi: 10.1098/rsos.170027 (PMC5451808; doi:10.1098/rsos.170027)
Supplement: ESM 7 [file rsos170027supp7.rtf]

# Code for analysis in R# Note the .csv files required are contained in ESM 3-5# The start of each analysis shows reading in the required file##### Figure 2 - Lionfish abundance, body size and weight changes######### Lionfish abundance, body size and weight with depth##### set uprm(list=ls())# Reading in datadf1<- read.csv(“ESM3.csv",               header=TRUE, colClasses='factor')# Selecting just lionfishdf1 <- df1[which(df1$species=='volitans'), ]# Creating dataframe for new lionfish observationsdepth <- c(5, 15, 25, 40)site <- unique(df1$site)transect <- c(2, 3, 4, 5)df2 <- expand.grid(site=site, depth=depth, transect=transect)temp <- expand.grid(site=c("RC", "RP", "TM"), depth=55, transect=transect)df2 <- rbind(df2, temp)temp <- expand.grid(site=c("TM"), depth=c(70, 85), transect=transect)df2 <- rbind(df2, temp)df2$abundance <- 0# adding lionfish depth data to lionfish dataframefor (i in 1:nrow(df1)){  row.no <- which(df2$site==df1$site[i] & df2$depth==df1$depth[i] & df2$transect==df1$transect[i])    df2$abundance[row.no] <- df2$abundance[row.no] + 1  } # closes the loop to add up lionfish abundance# option to select just RC, RP and TM lionfish abundance datadf3 <- df2 #[which(df2$site=="RC" | df2$site=="RP" | df2$site=="TM"), ] # turned off# Calculating the mean abundance per depth at each sitedf4 <- aggregate(abundance ~ site + depth, data=df3, FUN=mean)# Standard error of the mean function to be usedsem <- function(x){  sqrt(var(x)/length(x))} # closes the sem function# Calculating mean abudnance per depthdf5 <- aggregate(abundance ~ depth, data=df4, FUN=mean)df6 <- aggregate(abundance ~ depth, data=df4, FUN=sem)df5$se <- df6$abundance# turning se into 95% confidence intervalsdf5$se <- df5$se *1.96##### Plotting####tiff("Fig 2 Lionfish abundance length weight hist.tiff", width = 4.5, height = 6.75, units = 'in', res = 300)par(mar=c(5, 5, 1, 0))par(oma=c(4, 0, 0 ,0))mat <- matrix(data=c(rep(1, 24), rep(c(2, 2, 2, 5, 5, 5), 2),                     rep(c(3, 3, 3, 6, 6, 6), 2), rep(c(4, 4, 4, 7, 7, 7), 2)), ncol=6, byrow=TRUE)layout(mat)barplot(df5$abundance, ylim=c(0, 3),        ylab=expression("Abundance (250m"^{2} ~")"),        xlab="Depth (m)", names.arg=df5$depth, las=1,        cex.lab=1.5, cex.axis=1.2, cex=1.4)                # Adding error barstotal <- nrow(df5)location <- seq(from=0.7, by=1.2, length.out=7)for (i in 1:total){  arrows(location[i], df5$abundance[i], location[i], df5$abundance[i]+df5$se[i], code=2, angle=90, length=0.1)  arrows(location[i], df5$abundance[i], location[i], df5$abundance[i]-df5$se[i], code=2, angle=90, length=0.1)  } # closes the i in total:1 looplegend("topleft", "A", bty="n", cex=1.5, adj=2)#### Second plots###df1 <- read.csv(“ESM4.csv")# Lionfish Lengthspar(mar=c(1, 6, 1, 0))df1$depth.zone <- as.factor(df1$depth.zone)# 0-25 mhist(df1$TL.mm[which(df1$depth.zone==1)],     axes=F, xlab="", ylab="", main="", freq=FALSE, breaks=10, xlim=c(0, 500), ylim=c(0, 0.005))axis(1, labels=FALSE)axis(2, las=2)legend("topleft", "B", bty="n", cex=1.5, adj=c(2, -0.5))legend("topleft", "0-25 m", bty="n", cex=1, adj=c(0.45, 1.5))legend("topleft", paste("n=", length(df1$TL.mm[which(df1$depth.zone==1)])), bty="n", cex=1, adj=c(0.45, 3))# 25-40 mhist(df1$TL.mm[which(df1$depth.zone==2)],     axes=F, xlab="", ylab="", main="", freq=FALSE, breaks=10, xlim=c(0, 500), ylim=c(0, 0.005))axis(1, labels=FALSE)axis(2, las=2)legend("topleft", "25-40 m", bty="n", cex=1, adj=c(0.45, 0.5))legend("topleft", paste("n=", length(df1$TL.mm[which(df1$depth.zone==2)])), bty="n", cex=1, adj=c(0.45, 2))mtext("Proportion of fish", side=2, line=4, outer=FALSE)# >40 mhist(df1$TL.mm[which(df1$depth.zone==3)],     axes=F, xlab="Length (mm)", ylab="", main="", freq=FALSE, breaks=10, xlim=c(0, 500), ylim=c(0, 0.005))axis(1, labels=TRUE)axis(2, las=2)legend("topleft", "40-72 m", bty="n", cex=1, adj=c(0.45, 0.5))legend("topleft", paste("n=", length(df1$TL.mm[which(df1$depth.zone==3)])), bty="n", cex=1, adj=c(0.45, 2))mtext("Length (mm)", side=1, outer=FALSE, line=2.5, las=1)p1 <- ks.test(df1$TL.mm[which(df1$depth.zone==1)], df1$TL.mm[which(df1$depth.zone==2)])p.adjust(p1$p.value, method="fdr", n=3)p2 <- ks.test(df1$TL.mm[which(df1$depth.zone==1)], df1$TL.mm[which(df1$depth.zone==3)])p.adjust(p2$p.value, method="fdr", n=3)p3 <- ks.test(df1$TL.mm[which(df1$depth.zone==2)], df1$TL.mm[which(df1$depth.zone==3)])p.adjust(p3$p.value, method="fdr", n=3)# Lionfish Weights# 0-25 mhist(df1$Weight.with.spines.g[which(df1$depth.zone==1)],     axes=F, xlab="", ylab="", main="", freq=FALSE, breaks=12, xlim=c(0, 1200), ylim=c(0, 0.0025))axis(1, labels=FALSE)axis(2, las=2)legend("topright", "C", bty="n", cex=1.5, adj=c(2, -0.5))legend("topright", "0-25 m", bty="n", cex=1, adj=c(0.25, 1.5))# 25-40 mhist(df1$Weight.with.spines.g[which(df1$depth.zone==2)],     axes=F, xlab="", ylab="", main="", freq=FALSE, breaks=12, xlim=c(0, 1200), ylim=c(0, 0.0025))axis(1, labels=FALSE)axis(2, las=2)legend("topright", "25-40 m", bty="n", cex=1, adj=c(0.25, 0.5))mtext("Proportion of fish", side=2, line=4, outer=FALSE)# >40 mhist(df1$Weight.with.spines.g[which(df1$depth.zone==3)],     axes=F, xlab="", ylab="", main="", freq=FALSE, breaks=12, xlim=c(0, 1200), ylim=c(0, 0.0025))axis(1, labels=TRUE)axis(2, las=2)legend("topright", "40-72 m", bty="n", cex=1, adj=c(0.25, 0.5))mtext("Weight (g)", side=1, outer=FALSE, line=2.5, las=1)p1 <- ks.test(df1$Weight.with.spines.g[which(df1$depth.zone==1)], df1$Weight.with.spines.g[which(df1$depth.zone==2)])p.adjust(p1$p.value, method="fdr", n=3)p2 <- ks.test(df1$Weight.with.spines.g[which(df1$depth.zone==1)], df1$Weight.with.spines.g[which(df1$depth.zone==3)])p.adjust(p2$p.value, method="fdr", n=3)p3 <- ks.test(df1$Weight.with.spines.g[which(df1$depth.zone==2)], df1$Weight.with.spines.g[which(df1$depth.zone==3)])p.adjust(p3$p.value, method="fdr", n=3)dev.off()#### Figure 3 - Lionfish maturity and gonad weight with depth###df1 <- read.csv(“ESM4.csv")df1$depth.zone <- as.factor(df1$depth.zone)df1$Gonad.weight.g <- as.numeric(as.character(df1$Gonad.weight.g))### Calculating the proportion of females at each maturity stage with depth##dff <- subset(df1, Sex=='F')dff <- table(dff$Maturity, dff$depth.zone)# turning into proportionstotals <- colSums(dff)for (i in 1:ncol(dff)){  dff[, i] <- dff[, i]/totals[i]} # closes the for loopdff.maturity <- dff# Calculating the proportion of immature lionfish at each depth# Immature lionfishimmature <- subset(df1, Sex=='I')immature <- data.frame(depth.zone=1:3, frequency = table(immature$depth.zone)[1:3], all = table(df1$depth.zone)[1:3])immature$proportion <- immature$frequency / immature$all#### Calulating mean gonad:body weight ratio for each depth###### Females##dff <- subset(df1, Sex=='F')dff$Gonad.weight.g <- dff$Gonad.weight.g/dff$Weight.with.spines.g# meanmean.Gonad.weight.g <- mean(dff$Gonad.weight.g[which(dff$depth.zone==1)], na.rm=TRUE)mean.Gonad.weight.g <- c(mean.Gonad.weight.g, mean(dff$Gonad.weight.g[which(dff$depth.zone==2)], na.rm=TRUE))mean.Gonad.weight.g <- c(mean.Gonad.weight.g, mean(dff$Gonad.weight.g[which(dff$depth.zone==3)], na.rm=TRUE))# sdsd.Gonad.weight.g <- sd(dff$Gonad.weight.g[which(dff$depth.zone==1)], na.rm=TRUE)sd.Gonad.weight.g <- c(sd.Gonad.weight.g, sd(dff$Gonad.weight.g[which(dff$depth.zone==2)], na.rm=TRUE))sd.Gonad.weight.g <- c(sd.Gonad.weight.g, sd(dff$Gonad.weight.g[which(dff$depth.zone==3)], na.rm=TRUE))# nn.Gonad.weight.g <- sum(!is.na(dff$Gonad.weight.g[which(dff$depth.zone==1)]))n.Gonad.weight.g <- c(n.Gonad.weight.g, sum(!is.na(dff$Gonad.weight.g[which(dff$depth.zone==2)])))n.Gonad.weight.g <- c(n.Gonad.weight.g, sum(!is.na(dff$Gonad.weight.g[which(dff$depth.zone==3)])))female.gonad <- cbind(mean.Gonad.weight.g, sd.Gonad.weight.g, n.Gonad.weight.g)### Males##dfm <- subset(df1, Sex=='M')dfm$Gonad.weight.g <- dfm$Gonad.weight.g/dfm$Weight.with.spines.g# meanmean.Gonad.weight.g <- mean(dfm$Gonad.weight.g[which(dfm$depth.zone==1)], na.rm=TRUE)mean.Gonad.weight.g <- c(mean.Gonad.weight.g, mean(dfm$Gonad.weight.g[which(dfm$depth.zone==2)], na.rm=TRUE))mean.Gonad.weight.g <- c(mean.Gonad.weight.g, mean(dfm$Gonad.weight.g[which(dfm$depth.zone==3)], na.rm=TRUE))# sdsd.Gonad.weight.g <- sd(dfm$Gonad.weight.g[which(dfm$depth.zone==1)], na.rm=TRUE)sd.Gonad.weight.g <- c(sd.Gonad.weight.g, sd(dfm$Gonad.weight.g[which(dfm$depth.zone==2)], na.rm=TRUE))sd.Gonad.weight.g <- c(sd.Gonad.weight.g, sd(dfm$Gonad.weight.g[which(dfm$depth.zone==3)], na.rm=TRUE))# nn.Gonad.weight.g <- sum(!is.na(dfm$Gonad.weight.g[which(dfm$depth.zone==1)]))n.Gonad.weight.g <- c(n.Gonad.weight.g, sum(!is.na(dfm$Gonad.weight.g[which(dfm$depth.zone==2)])))n.Gonad.weight.g <- c(n.Gonad.weight.g, sum(!is.na(dfm$Gonad.weight.g[which(dfm$depth.zone==3)])))male.gonad <- cbind(mean.Gonad.weight.g, sd.Gonad.weight.g, n.Gonad.weight.g)# Calculating standard errorcolnames(female.gonad) <- c("mean", "sd", "n")colnames(male.gonad) <- c("mean", "sd", "n")female.gonad <- as.data.frame(female.gonad)male.gonad <- as.data.frame(male.gonad)female.gonad$se <- female.gonad$sd / sqrt(female.gonad$n)male.gonad$se <- male.gonad$sd / sqrt(male.gonad$n)###### Plotting#####tiff("Fig 3 Lionfish maturity gonad.tiff", width = 6, height = 7, units = 'in', res = 300)par(cex.axis=1.2, cex.lab=1.2)par(mar=c(3, 4, 1, 1))par(oma=c(2, 0, 0, 0))par(mfrow=c(2,2))# Female Maturitypar(mar=c(3, 4, 4, 1))colour <- gray.colors(5, start=0.1, end=1)barplot(as.matrix(dff.maturity), ylim=c(0, 1),        las=1, cex.lab=1, col=colour,        names.arg=c("0 - 25", "25 - 40", "40 - 72"))mtext("Proportion of females", 2, line=2.5, cex=1)par(xpd=TRUE)labels <- 1:5legend(-0.1, 1.16, legend=labels, text.width=0.1,       fill=colour, ncol=5, cex=1.4, bty='n', x.intersp=0.3)legend("topleft", "A", bty='n', cex=1.4, adj=c(2, -3.8))par(xpd=FALSE)# Plotting immature lionfishpar(mar=c(3, 4, 1, 1))barplot(immature$proportion, names.arg=c("0 - 25", "25 - 40", "40 - 72"),        xlab="", axes=FALSE, ylim=c(0, 0.2))axis(2, las=2, at=c(0, 0.05, 0.1, 0.15), labels=c(0, 0.05, "0.10", 0.15))legend("topleft", "B", bty='n', cex=1.4, adj=c(2, -0.1))mtext("Proportion immature", side=2, line=3.5, cex=0.9)# Gonad weight - Femalebarplot(female.gonad$mean, ylim=c(0, 0.06),        names.arg=c("0 - 25", "25 - 40", "40 - 72"), ylab="",        main="", las=1)positions <- seq(0.7, 3.9, 1.2)for (i in 1:nrow(female.gonad)){  arrows(x0=positions[i], y0=female.gonad$mean[i],         x1=positions[i], y1=female.gonad$mean[i] - (1.96* female.gonad$se[i]),         angle=90, length=0.12)    arrows(x0=positions[i], y0=female.gonad$mean[i],         x1=positions[i], y1=female.gonad$mean[i] + (1.96 * female.gonad$se[i]),         angle=90, length=0.12)  } # closes the error bar loopmtext("Female gonad:body weight ratio", 2, line=3, cex=0.9)legend("topleft", "C", bty='n', cex=1.4, adj=c(2, -0.5))mod4 <- aov(Gonad.weight.g ~ depth.zone, data=dff)summary(mod4)TukeyHSD(mod4)text(x=positions[c(1, 2, 2, 3)], y=(female.gonad$mean[c(1, 2, 2, 3)] + (female.gonad$se[c(1, 2, 2, 3)]*1.96) + c(0.005, 0.005, 0.01, 0.005)),     labels=c(expression(italic("A")),              expression(italic("A")),              expression(italic("B")),              expression(italic("B"))))# Gonad Weight Mpar(mar=c(3, 4, 1, 1))barplot(male.gonad$mean, ylim=c(0, 0.001),        names.arg=c("0 - 25", "25 - 40", "40 - 72"), ylab="",        main="", las=1, axes=F)axis(2, las=3, at=c(0, 0.0002, 0.0004, 0.0006, 0.0008, 0.0010),     labels=c(0, "0.0002", "0.0004", "0.0006", "0.0008", "0.0010"))positions <- seq(0.7, 3.9, 1.2)for (i in 1:nrow(male.gonad)){  arrows(x0=positions[i], y0=male.gonad$mean[i],         x1=positions[i], y1=male.gonad$mean[i] - (1.96* male.gonad$se[i]),         angle=90, length=0.12)    arrows(x0=positions[i], y0=male.gonad$mean[i],         x1=positions[i], y1=male.gonad$mean[i] + (1.96* male.gonad$se[i]),         angle=90, length=0.12)  } # closes the error bar loopmtext("Male gonad:body weight ratio", side=2, line=3, cex=0.9)legend("topleft", "D", bty='n', cex=1.4, adj=c(2, -0.5))mod6 <- aov(Gonad.weight.g ~ depth.zone, data= dfm)summary(mod6)TukeyHSD(mod6)mtext("Depth band (m)", 1, line=0.5, outer=TRUE)dev.off()# Proportion of immature males and females# Males(table(dfm$Maturity)[1])/sum(table(dfm$Maturity)[1:2]) * 100# Females(table(dff$Maturity)[1])/sum(table(dff$Maturity)[1:5]) * 100# Proportion of immature fish that could be sexeddfi <- subset(df1, Maturity==1)sum(table(dfi$Sex)[c(2, 4)])/sum(table(dfi$Sex)) * 100#### Figure 4 - Lionfish condition and diet with depth###library(vegan)# reading in the stomach datadf1 <- read.csv(“ESM6.csv")# reading in the broader dissection datadf2 <- read.csv(“ESM4.csv")# sorting data typesdf2$depth.zone <- as.factor(df2$depth.zone)df2$Lionfish.ID <- as.character(df2$Lionfish.ID)df1$lionfish.ID <- as.character(df1$lionfish.ID)#### A Calculating the proprtion of body fat for each lionfish###df2$prop.fat <- df2$Fat.g/df2$Weight.with.spines.g# Checking whether there's any correlation between proportion of body fat and sizemod1 <- cor.test(df2$TL.mm, df2$prop.fat, na.rm=TRUE, method="pearson")summary(mod1)# Checking for any correlation between gonad weight and proportion of fat + maturity for femalesdf3 <- df2[which(df2$Sex=="F"), ]df3$Gonad.weight.g <- as.numeric(as.character(df3$Gonad.weight.g))mod2 <- lm((Gonad.weight.g)^(1/4) ~ prop.fat + Maturity,           data=df3)summary(mod2)### B Calculating the proprtion of lionfish with stomach food### Convert the presence of food item into 1 and empty stomachs into 0df1$food.present <- df1$item# releveling factor, so that "Empty" stomachs get factor level 1 and all other factor levels >1df1$food.present <- as.numeric(relevel(df1$food.present, "Empty"))# Identifying each row represnting an inspected lionfish with an empty stomach and giving them 0df1$food.present[which(df1$food.present==1)] <- 0# Giving all lionfish stomach food items the value 1df1$food.present[which(df1$food.present>1)] <- 1# calculating the total number of items in the lionfish stomachdf3 <- aggregate(food.present ~ lionfish.ID, data=df1, sum)# Converting to 1 to indicate presence of itemsdf3$food.present[which(df3$food.present>1)] <- 1# Adding the depth band to the data frame# requires cross checking each lionfish ID number between the two datasheetsdf3$depth.zone <- NAfor (i in 1:nrow(df3)){  row.no <- which(df2$Lionfish.ID==df3$lionfish.ID[i])  if(length(row.no) == 1)  {    df3$depth.zone[i] <- df2$depth.zone[row.no]  } # closes if statment} # closes the for loop# Proportion of stomaches with food inprop.with.food <- table(df3$food.present, df3$depth.zone)[2, ]/  colSums(table(df3$food.present, df3$depth.zone))mod3 <- glm(food.present ~ as.factor(depth.zone), data=df3, family=binomial(link=logit))summary(mod3)# function to back transform logit model outputsexpit <- function(x){  e <- exp(1)  (e^x)/(1+(e^x))}#### C Proportion of different groups making up stomach contents###df4 <- as.data.frame.matrix(table(df1$lionfish.ID, df1$item))# Grouping all low abundance invertebrates into one groupdf4$Other.Invert <- rowSums(df4[, c("Cephalopod", "Gastropod", "Invertebrate", "Isopod", "Urchin")])# grouping lobsters and shrimp togetherdf4$Shrimp <- rowSums(df4[, c("Shrimp", "Lobster")])# Final data framedf5 <- df4[, c("Fish", "Shrimp", "Crab", "Other.Invert", "Algae")]df5$depth.zone <- NA# Adding in depth bandsfor (i in 1:nrow(df5)){  row.no <- which(df2$Lionfish.ID==rownames(df5)[i])   if(length(row.no) == 1)  {    df5$depth.zone[i] <- df2$depth.zone[row.no]  } # closes the if statement} # closes the adding depth band loop# Turning abundances into proportions# New empty dataframe for resultsdf6 <- df5[1, ]df6 <- df6[-1, ]# Calculating the proportion of each group at each depth zonefor (i in 1:3){  temp <- subset(df5, depth.zone==i)  totals <- colSums(temp[, 1:5])  prop <- totals/sum(totals)  row.no <- nrow(df6) + 1  df6[row.no, ] <- c(prop, i)}#### D NMDS plot of fish community comprising stomach content#### Selecting just the fish which have been identified from the stomachsdf7 <- df1[which(df1$item=="Fish" & (is.na(df1$family)==FALSE)), ]# releveling familydf7$family <- droplevels(df7$family)# Rearranging so that each family is a columndf7 <- as.data.frame.matrix(table(df7$lionfish.ID, df7$family))# Adding in depth bands and lengthdf7$depth.zone <- NAfor (i in 1:nrow(df7)){  row.no <- which(df2$Lionfish.ID==rownames(df7)[i])   if(length(row.no) == 1)  {    df7$depth.zone[i] <- df2$depth.zone[row.no]    df7$TL.mm[i] <- df2$TL.mm[row.no]      } # closes the if statement} # closes the adding depth band loop# removing any fish that do not have a depth associated with themdf7 <- df7[which(is.na(df7$depth.zone)==FALSE), ]# Allocating a body length group to fish# VS - very small <150 mm# S - small 150 - 250# M - medium 250-350 mm# L - large >350 mmdf7$TL.mm[which(df7$TL.mm < 150)] <- 1df7$TL.mm[which(df7$TL.mm >= 150 & df7$TL.mm < 250)]  <- 2df7$TL.mm[which(df7$TL.mm >= 250 & df7$TL.mm < 350)]  <- 3df7$TL.mm[which(df7$TL.mm >= 350)] <- 4# Taking mean abundance of each depth band and body sizedf8 <- colSums(df7[which(df7$depth.zone==1 & df7$TL.mm==1), ] )df8 <- rbind(df8, colSums(df7[which(df7$depth.zone==1 & df7$TL.mm== 2), ] ))df8 <- rbind(df8, colSums(df7[which(df7$depth.zone==1 & df7$TL.mm== 3), ] ))df8 <- rbind(df8, colSums(df7[which(df7$depth.zone==1 & df7$TL.mm== 4), ] ))df8 <- rbind(df8, colSums(df7[which(df7$depth.zone==2 & df7$TL.mm== 1), ] ))df8 <- rbind(df8, colSums(df7[which(df7$depth.zone==2 & df7$TL.mm== 2), ] ))df8 <- rbind(df8, colSums(df7[which(df7$depth.zone==2 & df7$TL.mm== 3), ] ))df8 <- rbind(df8, colSums(df7[which(df7$depth.zone==2 & df7$TL.mm== 4), ] ))df8 <- rbind(df8, colSums(df7[which(df7$depth.zone==3 & df7$TL.mm== 1), ] ))df8 <- rbind(df8, colSums(df7[which(df7$depth.zone==3 & df7$TL.mm== 2), ] ))df8 <- rbind(df8, colSums(df7[which(df7$depth.zone==3 & df7$TL.mm== 3), ] ))df8 <- rbind(df8, colSums(df7[which(df7$depth.zone==3 & df7$TL.mm== 4), ] ))# sorting the output into a dataframedf8 <- (as.matrix(df8))row.names(df8) <- 1:nrow(df8)df8 <- as.data.frame(df8)# updating the descriptor columnsdf8$depth.zone <- c(1, 1, 1, 1, 2, 2, 2, 2, 3, 3, 3, 3)df8$TL.mm <- rep(1:4, 3)# removing rows with no lionfish with stomach fish recordedrowSums(df8[, 1:19])df8 <- df8[c(-1, -9), ]# Calculating dissimilaritiesfish.matrix <- vegdist((df8[, 1:(ncol(df8)-2)])^(1/4))NMDS <- metaMDS(fish.matrix, k=2)###### Plotting####tiff("Fig 4 Lionfish condition feeding.tiff", width = 6, height = 6, units = 'in', res = 300)par(mfrow=c(2, 2))par(mar=c(3, 4, 1, 1))#### A Proportion body fat###plot(prop.fat ~ depth.zone, data=df2, ylim=c(0, 0.05),     names=c("0 - 25", "25 - 40", "40 - 72"), xlab="", ylab="",     frame=F, las=1, cex.axis=0.8)mtext("Depth band (m)", 1, line=2.2, cex=0.8)mtext("Proportion of body fat", 2, line=2.5, cex=0.8)points(x=c(1, 2, 3), y=tapply((df2$prop.fat), df2$depth.zone, mean, na.rm=T),       pch=15, cex=1)mod4 <- aov(prop.fat ~ depth.zone, data=df2)TukeyHSD(mod4)# Meantapply((df2$prop.fat), df2$depth.zone, mean, na.rm=T)# Standard errortemp <- df2[which(is.na(df2$depth.zone)==FALSE), ]temp <- temp[which(is.na(temp$prop.fat)==FALSE), ]tapply((temp$prop.fat), temp$depth.zone, sd) /   sqrt(tapply((temp$prop.fat), temp$depth.zone, length))text(x=c(1, 2, 3), y=c(0.046, 0.046, 0.046), labels=c(expression(italic("A")),                                                      expression(italic("A")),                                                      expression(italic("B"))))legend("topleft", "A", bty="n", cex=1.5, adj=c(2, -0.5))#### B Proportion of stomaches with food in###barplot(prop.with.food, ylim=c(0, 1),        names=c("0 - 25", "25 - 40", "40 - 72"), xlab="", ylab="",        cex.names=0.85, las=1)mtext("Depth band (m)", 1, line=2.2, cex=0.8)mtext("Proportion of lionfish with food in stomach", 2, line=2.5, cex=0.8)legend("topleft", "B", bty="n", cex=1.5, adj=c(2, -0.5))#### C Proportion of different groups making up stomach contents###par(mar=c(4, 4, 4, 1))colour <- gray.colors(4, start=0.1, end=1)colour <- c(colour, rep(colour[length(colour)], 2))barplot(t(as.matrix(df6[, 1:5])), ylim=c(0, 1),        las=1, cex.names=0.85, col=colour,        names.arg=c("0 - 25", "25 - 40", "40 - 72"))mtext("C", side=3, line=1.8, cex=1.2, adj=-0.15)mtext("Proportion of stomach contents", 2, line=2.5, cex=0.8)mtext("Depth band (m)", 1, line=2.2, cex=0.8)par(xpd=TRUE)labels <- colnames(df6)[1:(ncol(df6)-2)]labels[4] <- "Other Invert"legend(0, 1.3, legend=labels, text.width=0.9,       fill=colour[1:4], ncol=2, cex=1, bty='n')par(xpd=FALSE)#### D NMDS plot of fish community comprising stomach content###par(mar=c(4, 4, 1, 1))plot(NMDS, type='n', ylim=c(-1, 0.5),     xlab="", ylab="")colour <- c("red", "orange", "blue", "purple")shape <- c(21, 22, 23)for (i in 1:nrow(df8)){  x <- NMDS$points[i, 1]  y <- NMDS$points[i, 2]    points(x=x, y=y, col="black", bg=colour[df8$TL.mm[i]], pch=shape[df8$depth.zone[i]], cex=1.5)} # closes the i loopmtext("NMDS1", 1, line=2.2, cex=0.8)mtext("NMDS2", 2, line=2.2, cex=0.8)legend("bottomleft", legend=c("<150 mm", "150-250 mm", "250-350 mm", ">350 mm",                              "0-25 m", "25-40 m", "40-72 m", "Stress = 0.12"),       col=c(colour, rep("black", 3), "white"), pch=c(rep(15, 4), shape), cex=0.9, ncol=2,       bty='n')lines(c(-1.5, 1.5), c(-0.5, -0.5))legend("topleft", "D", bty="n", cex=1.5, adj=c(2, -0.5))dev.off()#### Table 1 - Proportion of fish by family recorded within all lionfish stomachs within each depth band#### Note - continues on from Figure 4 analysis# Taking mean abundance of each depth band and body sizedf9 <- colSums(df7[which(df7$depth.zone==1), ] )df9 <- rbind(df9, colSums(df7[which(df7$depth.zone==2), ] ))df9 <- rbind(df9, colSums(df7[which(df7$depth.zone==3), ] ))df9 <- (as.matrix(df9))row.names(df9) <- 1:nrow(df9)df9 <- as.data.frame(df9)df9 <- df9[, -c(20, 21)]df9 <- t(df9)# Turning each column into proportionsfor (i in 1:ncol(df9))  {  total <- sum(df9[, i])  for(j in 1:nrow(df9))    {    df9[j, i] <- df9[j, i]/total    } # closes the j loop  } # closes the i loopwrite.csv(df9, file="Table 1.csv")#### Table 2 - ANCOVA results testing differences in lionfish alert distance with depth on Utila and Tela.#### reading in the alert datadf1 <- read.csv(“ESM5.csv")df1$alert.distance.cm[which(df1$alert.distance.cm < 1)] <- 1### Running model using depths zones##df1$depth.zone[which(df1$depth.m >= 40)] <- 3df1$depth.zone[which(df1$depth.m < 25)] <- 1# removing intermediate depthsdf1 <- df1[(is.na(df1$depth.zone)==FALSE), ]df1$depth.zone <- paste(df1$region, df1$depth.zone, sep="_")mod1 <- lm(log(alert.distance.cm) ~ depth.zone + total.length.cm,            data=df1)summary(mod1)mod2 <- aov(log(alert.distance.cm) ~ depth.zone,           data=df1)summary(mod2)TukeyHSD(mod2)# Median differencemedian(df1$alert.distance.cm[which(df1$depth.zone=="Utila_1")])median(df1$alert.distance.cm[which(df1$depth.zone=="Utila_3")])median(df1$alert.distance.cm[which(df1$depth.zone=="Tela_1")])predict.A <- data.frame(depth.zone=c("Utila_1", "Utila_1"), total.length.cm=c(10, 30))predict.A$alert <- predict(mod1, newdata=predict.A, type="response" )predict.Aexp(predict.A$alert)
