# Supplementary material for: Depth-dependent effects of culling—do mesophotic lionfish populations undermine current management?
Source: R Soc Open Sci. 2017 May 24;4(5):170027. doi: 10.1098/rsos.170027 (PMC5451808; doi:10.1098/rsos.170027)
Supplement: ESM 8 [file rsos170027supp8.docx]

**ESM 8 Results of pairwise Kolmogorov-Smirnov** **tests to compare lionfish (A) length and (B) weight distributions shown in Figure 2B and 2C.** Pairwise two-sample Kolmogorov-Smirnov tests were used to compare distributions, and p-values adjusted to control for multiple comparisons based on the false-discovery rate (see: Benjamini & Hochberg, 1995).

(A) Lionfish length distributions:

| Comparison | Kolmogorov-Smirnov D | Adjusted p-value |
| --- | --- | --- |
| 0-25 m vs 25-40 m | 0.14 | 0.013 |
| 0-25 m vs 40-72 m | 0.20 | <0.001 |
| 25-40 m vs 40-72 m | 0.23 | <0.001 |

(B) Lionfish weight distributions:

| Comparison | Kolmogorov-Smirnov D | Adjusted p-value |
| --- | --- | --- |
| 0-25 m vs 25-40 m | 0.29 | <0.001 |
| 0-25 m vs 40-72 m | 0.32 | <0.001 |
| 25-40 m vs 40-72 m | 0.13 | 0.462 |
