# Supplementary material for: Depth-dependent effects of culling—do mesophotic lionfish populations undermine current management?
Source: R Soc Open Sci. 2017 May 24;4(5):170027. doi: 10.1098/rsos.170027 (PMC5451808; doi:10.1098/rsos.170027)
Supplement: ESM 9 [file rsos170027supp9.docx]

ESM 9. ANCOVA results for the effect of proportion of body fat, maturity and depth on female lionfish gonad weight. Lionfish gonad weight was fourth root transformed to meet ANCOVA assumptions. Depth was fitted as a categorical variable, with the intercept representing Depth: 0-25 m, and other depth values given as a difference from the intercept. Maturity and Lionfish Weight estimates represent slopes of the model.

|  | Estimate | Standard Error | *t* value | *P* |
| --- | --- | --- | --- | --- |
| Intercept | 0.282 | 0.06 | 4.64 | <0.001 |
| Proportion of fat | -9.737 | 3.14 | -3.10 | 0.002 |
| Maturity | 0.235 | 0.02 | 11.89 | <0.001 |
| Lionfish Weight | 0.002 | 0.00 | 8.54 | <0.001 |
| Depth: 25-40 m | 0.107 | 0.05 | 2.08 | 0.040 |
| Depth: 40-72 m | 0.132 | 0.05 | 2.88 | 0.004 |
