# Supplementary material for: Depth-dependent effects of culling—do mesophotic lionfish populations undermine current management?
Source: R Soc Open Sci. 2017 May 24;4(5):170027. doi: 10.1098/rsos.170027 (PMC5451808; doi:10.1098/rsos.170027)
Supplement: ESM 10 [file rsos170027supp10.docx]

ESM 10. GLM results for differences in the proportion of lionfish stomachs containing food with depth. Binomial GLM was fitted with a logit link function. The intercept represents the shallow group (0–25 m), with other estimates given as the difference from the intercept. Residual deviance: 1135.4 on 1201 degrees of freedom.

|  | Estimate | Standard Error | *z* value | *P* |
| --- | --- | --- | --- | --- |
| Intercept | 1.55 | 0.08 | 18.28 | <0.001 |
| 25 – 40 m | 0.06 | 0.25 | 0.25 | 0.806 |
| 40 – 72 m | -0.45 | 0.23 | -1.89 | 0.059 |
